# Parental Mental Well-Being and Frequency of Adult-Child Nature Visits: The Mediating Roles of Parents’ Perceived Barriers

**DOI:** 10.3390/ijerph18136814

**Published:** 2021-06-25

**Authors:** Jasmine Gustafsson, Ann Ojala, Pauliina Hiltunen, Elina Engberg, Annika Wiklund-Engblom, Nea Törnwall, Eva Roos, Carola Ray

**Affiliations:** 1 Public Health Research Program, Folkhälsan Research Center, FI-00250 Helsinki, Finland; pauliina.hiltunen@helsinki.fi (P.H.); elina.engberg@helsinki.fi (E.E.); eva.roos@folkhalsan.fi (E.R.); carola.ray@folkhalsan.fi (C.R.); 2 Natural Resources Institute Finland (Luke), FI-00791 Helsinki, Finland; ann.ojala@luke.fi; 3 Department of Psychology and Logopedics, University of Helsinki, FI-00014 Helsinki, Finland; 4 Folkhälsans Förbund, FI-65100 Vaasa, Finland; Annika.Wiklund-Engblom@folkhalsan.fi (A.W.-E.); Nea.Tornwall@folkhalsan.fi (N.T.); 5 Department of Public Health, University of Helsinki, FI-00014 Helsinki, Finland; 6 Department of Food Studies, Nutrition and Dietetics, Uppsala University, 752 36 Uppsala, Sweden; 7 Department of Food and Nutrition, University of Helsinki, FI-00014 Helsinki, Finland

**Keywords:** nature visits, parental factors, barriers to visiting nature, early childhood, mediation analysis

## Abstract

Regular access to green space has been shown to provide several health benefits for children. However, children today spend less time outdoors. Thus, it has become important to understand what drives and limits children’s activities in nature. Based on a Finnish online survey of 1463 parents of children aged 2–7 conducted in 2019, the current study examined parents’ perceived barriers to visiting nature with their children. It also examined how parental mental well-being is related to families’ frequency of nature visits, and whether this association is mediated by different categories of parents’ perceived barriers. Eleven out of 12 barriers were largely perceived by parents as reasons that did not prevent them from visiting nature with their children. Next, factor analysis indicated a three-factor solution to the barriers. The results of a multiple mediation analysis showed that better parental mental well-being was associated with more frequent adult-child nature visits, and this relationship was partially mediated by a “lack of competence and logistics” and a “lack of time and interest”, but not by “insecurity and fear”. The results indicated that parents with poor mental well-being were more likely to perceive barriers to visiting nature, which in turn appeared to be related to a higher likelihood of having children who visited nature less frequently.

## 1. Introduction

Regular access to natural environments and interacting with nature have been shown to provide a great number of health benefits for children [1,2]. For example, in a review by McCormick [1], regular access to green space was associated with improved mental well-being, overall health and cognitive development of children aged between 0 to 18 years. Moreover, another review by Tillmann et al. [2] found that interacting with nature was related to increased overall mental health, improvements in symptoms of attention deficit hyperactivity disorder and attention deficit disorder (ADD/ADHD), reduced stress, higher resilience, and improved health-related quality of life among children and adolescents younger than 18 years. In terms of physical health outcomes, exposure to green spaces has been linked with lower blood-pressure [3], longer sleep [4], improvement in motor fitness [5], and a lower prevalence of overweight/obesity and sedentary behavior [6] among children. In addition, the time children spend outdoors has been found to be more strongly related to physical activity than time spent indoors [7]. However, despite the potential health benefits of natural environments, children today spend less time outdoors interacting with nature [8,9]. At the same time, in several countries, a great number of children do not meet physical activity recommendations [10,11], which could partly be due to excessive use of media [12]. As encouraging young people to spend more time in nature could be a cost-effective way to increase their levels of physical activity and reduce their sedentary behavior, it has become important to understand what drives and limits children’s activities in nature. Since children spend more time in nature and green spaces with adults than without them [13], children’s opportunities to engage with and be physically active in nature are assumed to at least partly depend on whether their parents or other responsible adults spend time with them in nature.

In the last few decades, there has been a growing interest in the vital links between parental mental well-being (i.e., positive aspects of mental health; [14]), parenting behaviors and children’s health behaviors. For example, a meta-analytical review by Lovejoy et al. [15] found that poor maternal mental well-being was associated with weakened parenting behaviors: higher levels of hostile/coercive behavior, and lower levels of engagement and positive social interactions. Aspects of parental mental health have also been linked with physical activity levels in children, although evidence of this has been mixed. Some cross-sectional and longitudinal studies have associated higher levels of depressive symptoms among parents, mainly mothers, with lower levels of physical activity among children from low-income families [16], with high-income mothers [17], and among girls in particular [18]. In contrast, other studies have found no associations between parental depression or parental mental well-being in terms of self-esteem and life satisfaction, and children’s physical activity [19,20]. While these studies have focused on physical activity levels among children, less is known about how parental mental well-being is related to children’s nature visits, which generally involve components of physical activity [7].

If parents perceive a high degree of barriers to taking their child out in nature, children’s opportunities to be physically active might be restricted [21]. In a Norwegian study, parents reported that the main barriers to visiting nature with their child were that the child was too busy during leisure time, and that the child spent too much time on homework. In addition, concerns about traffic were perceived as a significant barrier [22]. Parents’ safety concerns seem to be an important reason why children spend less time outdoors [23,24]. A review by Carver et al. [25] revealed that parental barriers in terms of perceptions of neighborhood safety were negatively related to children’s and adolescents’ physical activity. For example, Weir et al. [26] found that higher parental anxiety about neighborhood safety was weakly associated with a lower amount of physical activity among 5- to 10-year-old children living in an inner-city environment in the US, but not among children living in a suburban environment. Moreover, a study of 10- to 12-year-old US children found that children of parents who perceived their neighborhood as unsafe for playing outside were less likely to use recreational facilities (e.g., public parks) and engaged less in physical activity than children whose parents perceived their neighborhood as very safe [27]. Parents’ perceptions of barriers, particularly in terms of safety concerns, could also be related to their mental health or well-being. For example, one previous study found that higher levels of anxiety were associated with higher overprotection (i.e., parenting behaviors that restrict a child’s exposure to perceived threats or harm) among mothers [28]. However, previous studies have not considered the potential link between parental mental well-being and parents’ perceived barriers to taking their child out in nature.

In sum, children today spend less time outdoors interacting with nature [8,9]. As spending time in nature is linked with a variety of health benefits (e.g., [1,2]), and families have been identified as playing a crucial role in influencing children’s nature-based activities (e.g., [13]), it has become important to understand which parental factors facilitate or hinder children’s nature visits. As argued above, poor parental mental well-being and higher levels of parents’ perceived barriers have previously been associated with lower levels of physical activity among children [15,17,18,25,27]. In addition, mothers reporting poor mental well-being have been demonstrated as tending to report being overly concerned about their children’s safety (i.e., overprotective) [28]. However, less is known about how parental mental well-being and parental barriers are related to adult-child nature activities. An understanding of these associations would provide valuable insights into how to promote children’s engagement in nature activities, and could be important for future parent- and family-focused health interventions.

**Figure 1:**
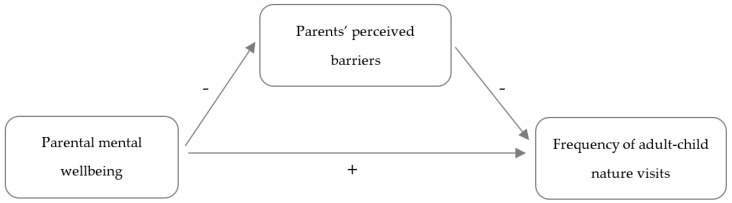
Hypothesized mediation model explaining associations between parental mental well-being, parents’ perceived barriers (mediator) and frequency of adult-child nature visits.

The first aim of this study was to examine parents’ perceived barriers to visiting nature with their child. The second aim was to examine the association between parental mental well-being and the frequency of families’ nature visits, and whether this association was mediated by different categories of parents’ perceived barriers. In the hypothesized mediation model, presented in Figure 1, better parental mental well-being was expected to be associated with a higher frequency of adult-child nature visits. Secondly, parents’ perceived barriers were expected to partially mediate this association, in a way that parents with poorer mental wellbeing would be more likely to perceive barriers to taking their child out in nature, which in turn would be associated with having children who are taken out in nature less frequently.

## 2. Materials and Methods

### 2.1. Participants and Procedure

In 2019, 1480 parents from different parts of Finland responded to an online cross-sectional survey and took part in the “Naturkraft” (Naturepower) project. Only parents with children meeting the age criteria of 2–7 years were included in this study, N = 1463 (approximately 99% of all participants). The main goal of the project was to gain new insights into the nature activities of families and the related parental and sociodemographic factors. The term “parental” referred to one of the child’s responsible adults, regardless of whether they had a genetic connection to the child. The survey was distributed through multiple channels consisting of networks of collaborative associations, organizations and day care centers, as well as Facebook groups targeted at local residents, parents and single parents. The survey was open from 29 April to 16 June 2019 in both Finnish and Swedish. The raw data of the study included a questionnaire consisting of a set of questions, of which only a few were included in this study. The University of Helsinki’s ethical review board in humanities and social and behavioral sciences granted ethical approval for this research (Statement 21/2019). Participation in the study was voluntary and participants provided their informed consent.

### 2.2. Measures

In the questionnaire, nature was defined as “natural outdoor and green areas such as forests, beaches, streams, fields and parks, but not own courtyards or built playgrounds”. Frequency of adult-child nature visits was assessed by asking parents the following question: “During the previous month, how often was at least one adult in the family out in nature with the child?” (1 = never, 2 = once, 3 = 2–3 times, 4 = once or twice a week, 5 = 3–4 times a week, and 6 = 5 times a week or more). This question was partially adapted from previous research [29,30].

Parental mental well-being was assessed using the Short Warwick Edinburgh Mental Well-being Scale (SWEMWBS) [31] which consists of seven items: “I’ve been feeling optimistic about the future”, “I’ve been feeling useful”, “I’ve been feeling relaxed”, “I’ve been dealing with problems well”, “I’ve been thinking clearly”, “I’ve been feeling close to other people”, and “I’ve been able to make up my own mind about things”. Respondents were asked to indicate how often they had experienced each statement in the last two weeks on a 5-point Likert scale (1 = none of the time, 2 = rarely, 3 = some of the time, 4 = often, and 5 = all of the time). The SWEMWBS was scored by first summing the scores for each of the seven items. The total raw scores were then transformed into metric scores using the SWEMWBS conversion table, with higher scores indicating better levels of mental well-being (range 7 to 35). Cronbach’s Alpha measure of internal consistency for the SWEMWBS in this study was 0.79. Permission to use the Finnish version of the scale was received from the Finnish Institute for Health and Welfare, and permission to use the Swedish version was received from the University of Warwick.

Parents’ perceived barriers were assessed by asking “Do the following reasons prevent you from visiting nature with your child?” followed by 12 items, which are presented in Figure 1. Parents rated each item on a 3-point scale (1 = no, 2 = to some extent, and 3 = very much). The items were partially adapted from previous research [22,29]. For further analysis, the items were grouped into three components by exploratory factor analysis. These were labelled “Lack of competence and logistics”, “Lack of time or interest”, and “Insecurity and fear”, and are presented in the results section.

Sociodemographic variables included the children’s age (years), children’s gender (1 = girl, 2 = boy), household type (1 = two-parent household, 2 = single-parent household), and parents’ perceived economic situation and the educational attainment of the parent who answered the questionnaire. The parents’ perceived economic situation was assessed by asking “How do you assess your family’s financial situation?” on a 5-point Likert scale (1 = very poor, 2 = fairly poor, 3 = decent, 4 = fairly good, and 5 = very good). Parental educational attainment was assessed on a 5-point scale (1 = primary school, 2 = vocational school, 3 = upper secondary school, 4 = polytechnic, lower university degree or institute degree, 5 = higher university degree or higher degree (licentiate/doctor)) and was recoded into three categories before the analyses (1 [low] = primary school, vocational school or upper secondary school graduate, 2 [medium]= bachelor’s degree or institute degree, and 3 [high] = master’s degree or higher).

### 2.3. Data Analysis

Statistical analyses were conducted using IBM SPSS Statistics 26.0 (IBM Corp, Armonk, NY, USA). Before all the analyses, the “Don’t know/Cannot say” options (<1% of responses in each variable) were coded as missing. The Nmiss-function was used before coding variables into mean scores, when only the parents who had answered at least 70% of the instrument’s questions were included in the preliminary and main analyses. Descriptive statistics were derived from means (M), standard deviations (SD) and frequencies (%). For the parents’ perceived barriers, an exploratory factor analysis was conducted using the principal component method with varimax rotation to examine whether these items enfolded several dimensions. The items were included in the factors if their loading was at least 0.40. Spearman’s rank correlation test was used to examine any possible association between the study and background variables.

Andrew Hayes Process Macro version 3.5 implemented in SPSS was used to test the hypothetical mediation model [32]. One single mediation analysis was run, which included all mediating variables in the same model. Bias-corrected bootstrapped 95% confidence intervals (CI) were produced of the mediation effects with 5000 bootstrapped samples generated by the bootstrapped sampling approach [32]. Only complete cases were included in the analysis (N = 1377). Children’s age, parents’ perceived economic situation, parental educational attainment, and household type were controlled for as covariates in the mediation analysis.

## 3. Results

### 3.1. Descriptive Statistics for Demographics and Key Variables

The descriptive statistics for demographic variables and the frequency of adult-child nature visits are summarized in Table 1. The majority of parents were 31 to 40 years old (67%), mothers (96%), and lived with a partner (89%). Of the children aged between two and seven years (M = 4.28, SD = 1.57), 49% were girls and 51% boys. The mean score of parental mental well-being was 23.56 (SD = 3.60). The mean score of the frequency of adult-child nature visits was 4.01 (SD = 1.22). Most parents reported that their children had been out in nature with at least one adult in the family once a week or more (67%) during the previous month, followed by one to three times a month (30%). Only 3% of the parents reported that their children had not been out in nature even once with an adult of the family during the previous month.

### 3.2. Identified Barriers Perceived by Parents

**Figure 2:**
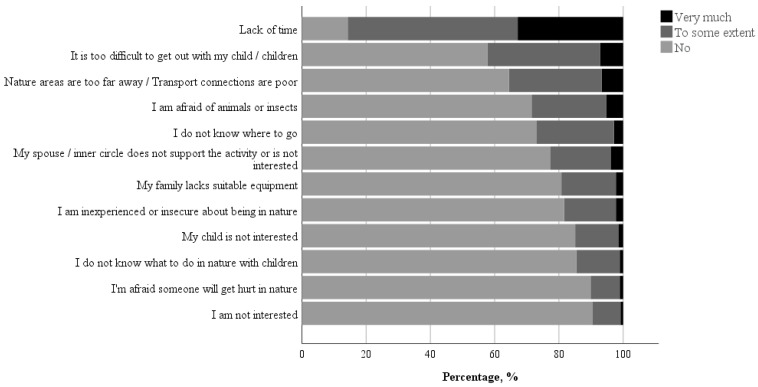
Perceived barriers to visiting nature as evaluated by parents in answer to the following question: “Do the following reasons prevent you from visiting nature with your child?” The data are presented as percentages of 12 items.

Of the 12 parents’ perceived barriers examined, 11 were largely perceived by parents as reasons that did not prevent them from visiting nature with their children (Figure 2). The most frequently reported barrier was “Lack of time”; 53% of parents reported this reason as preventing them from visiting nature with their child to some extent, and 33% of parents reported this reason as preventing them a great deal. Other barriers that parents more frequently perceived as reasons that prevented them at least to some extent from visiting nature with their children included it being too difficult to get out with their children, followed by nature areas being too far away or transport connections being poor, parents being afraid of animals or insects, and not knowing where to go. Items that were less frequently reported as barriers were related to the parents not being interested, followed by being afraid someone would get hurt in nature, and not knowing what to do in nature.

### 3.3. Exploratory Factor Analysis of Parents’ Perceived Barriers

For further analysis, the items of parents’ perceived barriers were grouped by an exploratory factor analysis. The factor structure and factor loadings of these items are presented in Table 2. The exploratory factor analysis suggested three main factors, each with eigenvalues above 1. The first factor, labelled “Lack of competence and logistics” (M = 1.27, SD = 0.35), included four items with loadings ranging from 0.41 to 0.81 and explained 16% of the variance. The second factor, labelled “Lack of time and interest” (M = 1.44, SD = 0.32), included five items with loadings ranging from 0.48 to 0.69 and explained 16% of the variance. The third factor, “Insecurity and fear” (M = 1.22, SD = 0.34), included three items and explained 15% of the variance with loadings ranging from 0.56 to 0.78. Composite scores were computed by averaging the scores within each subscale. Cronbach’s alpha values for the subscales were α = 0.62 for factor 1, α = 0.55 for factor 2, and α = 0.57 for factor 3. In the sections below, these three factors are referred to as “categories of parents’ perceived barriers”.

### 3.4. Correlations among Study Variables

Spearman’s rank correlation coefficients are presented in Table 3. The frequency of adult-child nature visits correlated positively with parental mental well-being, and negatively with all three categories of parents’ perceived barriers (*p* < 0.001). Of the background variables, child gender was not significantly associated with any study variables and was thus not controlled for in subsequent analyses.

### 3.5. Parents’ Perceived Barriers as Mediating Variables in the Relationship between Parental Mental Well-Being and Frequency of Adult-Child Nature Visits

**Figure 3:**
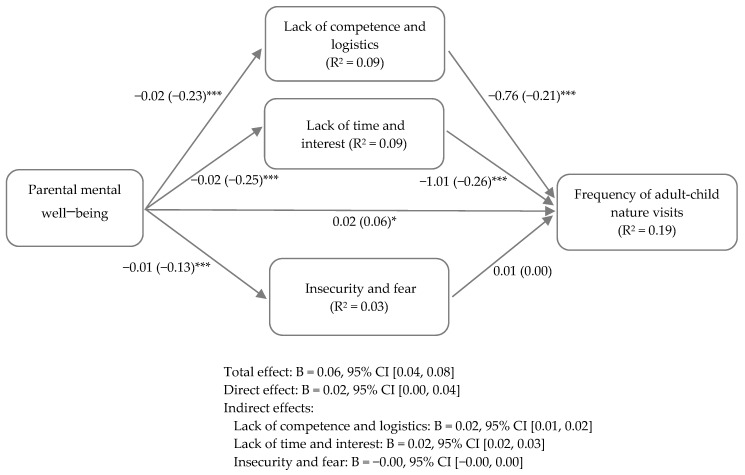
Parents’ perceived barriers as mediating variables in relationship between parental mental well-being and frequency of adult-child nature visits. Unstandardized regression coefficients (B) with standardized regression coefficients (β) in parentheses. All mediating variables included in the same model. Adjusted for background variables of child age, household type, parental educational attainment, and parents’ perceived economic situation. *** *p* < 0.001; * *p* < 0.05.

As shown in the multiple mediation model in Figure 3, when we controlled for background variables, we found a direct positive relationship between parental mental well-being and the frequency of adult-child nature visits (*p* < 0.05), meaning that better parental mental well-being was related to a higher frequency of adult-child nature visits. Parental mental well-being was also negatively associated with parents’ perceived barriers in all three barrier categories (*p* < 0.001), meaning that worse parental mental well-being was related to parents perceiving these statements as barriers to visiting nature with their children. Two of the parents’ perceived barrier categories—“Lack of competence and logistics” and “Lack of time and interest”—were negatively related to frequency of adult-child nature visits (*p* < 0.001), indicating that perceiving these statements as barriers to visiting nature was associated with a lower frequency of adult-child nature visits. The “Insecurity and fear” barrier category was not significantly related to the frequency of adult-child nature visits (*p* > 0.05).

The multiple mediation model was significant and explained 19% of the variance in the frequency of adult-child nature visits (R^2^ = 0.19, F(8, 1368) = 40.13, *p* < 0.001). Among the evaluated mediators, two of the parents’ perceived barriers categories—“Lack of competence and logistics” and “Lack of time and interest”—partially and significantly mediated the association between parental mental well-being and the frequency of adult-child nature visits (*p* < 0.001; indirect effects presented in Figure 3). However, the “Insecurity and fear” barrier category did not significantly mediate this association (*p* > 0.05). For the regression coefficient values of the background variables included in the mediation model, see Supplementary Table S1.

## 4. Discussion

The present study examined parents’ perceived barriers to visiting nature with their children. This was also the first time that the association between parental mental well-being and the frequency of adult-child nature visits in families was studied, and whether different categories of parents’ perceived barriers mediate this association. The goal of the study was to gain a deeper understanding of how various parental factors relate to nature visit frequency in families, as this could be of importance in future parent- and family-focused health interventions. We found that 11 out of the 12 single barriers were largely perceived by parents as reasons that did not prevent them from visiting nature with their children. Factor analysis indicated a three-factor solution for the barriers. Mediation analysis showed that better parental mental well-being was directly associated with more frequent adult-child nature visits when background variables were controlled for. We also found that two of the barrier categories—”Lack of competence and logistics” and “Lack of time and interest”—significantly mediated the association between parental mental well-being and the frequency of adult-child nature visits. However, “Insecurity and fear” did not significantly mediate this association.

Although most of the examined barriers were largely perceived by parents as reasons that did not prevent them from visiting nature with their children, some of them were perceived as more important than others. The most frequently reported barrier was lack of time, followed by difficulty getting out with the child, nature areas being too far away, or transport connections being poor, and parents being afraid of animals or insects and not knowing where to go. Some of these results are in line with those of a previous Norwegian study in which parents generally perceived a low degree of barriers and that time pressure in children’s leisure time was the main barrier to visiting nature with their children [22]. Thus, time management advice and scheduling free-time activities could possibly help families reduce obstacles to visiting nature.

The results of the mediation analysis make an important contribution as they show that parents with poor mental well-being were more likely to perceive barriers in two categories, which in turn was related to a higher likelihood of having children who less frequently visited nature with an adult. Among our hypothesized mediators of the association between parental mental well-being and the frequency of adult-child nature visits, “Lack of time and interest” appeared to be the main mediator, followed by “Lack of competence and logistics”. Our mediation model explained about 19% of the association, indicating that other mediators may also be involved. Future research could explore these further. We also found that parents with poor mental well-being were more likely to perceive barriers in all three barrier categories. As these associations have not been studied before, we were unable to compare the results of the mediation model with previous findings.

These findings could have implications for future intervention strategies. Further studies need to explore whether a causal link exists between the variables, and if this is the case, intervention strategies could focus on screening parents for poor mental well-being and offer them support and treatment. This, in turn, could possibly increase adult-child nature visits both directly and indirectly through lower levels of perceived barriers. Another option could be to arrange nature-based interventions aimed at both improving parental well-being and supporting children’s opportunities to get out into nature with their parents. For instance, this could be attained through low-cost guided group activities for families. Parents would possibly overcome barriers as the guide could provide instructions on where to go and what to do outdoors, and they would have the opportunity to recover in nature while simultaneously being able to interact with and receive support from other parents. Their children could also benefit from these group activities in several ways, as they would have the opportunity to spend time outdoors, improve their relationship with nature, play with peers, and increase their physical activity. Smartphone apps could also be utilized. For example, a previous intervention aimed at increasing the connection with nature and well-being among adults used a smartphone app for 30 days, and found that the well-being of participants who had been using the app improved significantly, and that participants with poorer well-being at baseline were even more likely to benefit from the intervention [33]. Parents could also be informed about apps for scheduling leisure-time activities, finding nature reserves or getting ideas for things to do in nature. This may help them perceive fewer barriers to going out into nature with their children (e.g., the iNaturalist application; [34]).

Among the limitations of the study were its cross-sectional design, meaning that we could make no causal interpretation of the relationships. Contrary to our hypothesized direction of associations, parents who spend more time in nature with their children may feel better mentally, and as a possible consequence, perceive fewer barriers. Another limitation is the use of self-reported data from only one source, mainly mothers (96%). On the other hand, the formulation of our question on nature visit frequency referred to at least one adult in the family, which could have been the mother, father or other adults in the family. Therefore, our results suggest that if one parent has poor mental well-being, their child is more likely to be taken out into nature less frequently by any adult in the family. In addition, the collected data did not clarify whether the recruited adults were biological parents, adoptive parents, or step-parents. The findings should be generalized with caution because the sample did not necessarily represent the population in general. Most parents in the sample had positive attitudes towards nature visits and self-selection bias may have occurred, leading parents who perceived nature activities as highly beneficial to be more willing to participate in this study. This could also be why to a large extent parents perceived the examined barriers as reasons that did not prevent them from visiting nature with their children. The high proportion of parents who appreciated nature activities in this sample could also be explained by a social desirability bias, causing respondents to answer in a manner that would be viewed favorably by others. It should also be noted that the mean score of the mental well-being measure (SWEMWBS) was somewhat lower in this study than in prior population-based samples in some Nordic countries [35].

The measures in this study may also have limitations. The nature visit frequency measure did not investigate the type of activities carried out during nature visits (e.g., walking, running, playing, climbing, swimming, or sitting), how engaged the child was, or for how long the activity lasted. Moreover, it did not explicitly clarify which adults of the family spent time in nature with the child. Finally, as the reliability of the parental barriers was not high, with values of Cronbach’s Alpha ranging from 0.55 to 0.62, the results should be interpreted with caution. Future research should continue to seek to understand the associations between parental mental well-being, perceived barriers and nature visit frequencies, and their implications for the health of both generations. For example, longitudinal research is needed, and researchers should collect data from multiple sources using multiple methods (observations, enrolment records, self- and other -reports) and further explore the types and intensity of the activities carried out in nature. Moreover, follow-up work is needed to assess the extent to which the results from this study are applicable to other cultures and all family structures, including those with same-sex parents or non-biological parents.

Despite these limitations, the present study has several important strengths, one of which is its large sample of participants (N = 1463) living in different parts of the country. Detailed information was collected on the parents’ barriers, and they were adjusted for a range of potential confounders of the associations between the study variables. The use of the SWEMWBS for measuring parental mental well-being was another strength, given that this scale has been widely used in population-based research [35,36,37] and has been shown to be a valid instrument for measuring mental well-being among adults in Denmark, Norway and Sweden [35,38]. In addition, our results highlight an interesting pathway between parental factors and the frequency of adult-child nature visits, and may inform future development of health-promoting activities.

This study was the first to examine whether parental mental well-being is related to the frequency of adult-child nature visits. Overall, most of the examined barriers were largely perceived by parents as reasons that did not prevent them from visiting nature with their children. Better parental mental well-being was associated with more frequent adult-child nature visits, and this relationship was partially mediated by “Lack of competence and logistics” and “Lack of time and interest”, but not by “Insecurity and fear”. As encouraging children to spend more time in nature could be a cost-effective way to increase their levels of physical activity and overall well-being, more work on deepening our understanding of the factors that may support or prevent their opportunities for nature activities is needed.

## 5. Conclusions

In a sample of 1463 participants, the present study found that most of the examined barriers were largely perceived by parents as reasons that did not prevent them from visiting nature with their children. Moreover, better parental mental well-being was associated with more frequent adult-child nature visits, and this relationship was partially mediated by “Lack of competence and logistics” and “Lack of time and interest”, but not by “Insecurity and fear”. More work on deepening our understanding of the factors that may support or prevent children’s opportunities for nature activities is needed.

## Figures and Tables

**Table 1:** Descriptive statistics for demographics of parents and frequency of adult-child nature visits.

Variable	*n*	%	*N* *
Parental characteristics			
Age of parent			1463
<31 years	264	18%	
31–40 years	991	68%	
>40 years	208	14%	
Questionnaire answered by			1463
Mother or stepmother	1408	96%	
Father or stepfather	44	3%	
Other guardian or adult	11	1%	
Household type (child living with one or two parents)			1433
Single-parent	152	11%	
Two-parent	1281	89%	
Parental educational attainment ^1^			1446
Low	410	28%	
Middle	632	44%	
High	404	28%	
Parents’ perceived economic situation			1455
Very poor	50	3%	
Fairly poor	173	12%	
Decent	666	46%	
Fairly good	453	31%	
Very good	113	8%	
Frequency of adult-child nature visits *^2^*			1463
Never	41	3%	
Once a month	101	7%	
2–3 times a month	340	23%	
Once or twice a week	490	33%	
3–4 times a week	305	21%	
5 times a week or more	186	13%	

Note. * indicates number of responses for each item. ^1^ low = primary school, vocational school or upper secondary school, middle = bachelor’s degree or institute degree, and high = master’s degree or higher. ^2^ in the last month.

**Table 2:** Items and factor loadings for parents’ perceived barriers.

Item	Factor 1 (Lack of Competence and Logistics)	Factor 2 (Lack of Time and Interest)	Factor 3 (Insecurity and Fear)
I do not know where to go	0.81		
I do not know what to do in nature with children	0.41		
Nature areas are too far away/Transport connections are poor	0.79		
My family lacks suitable equipment	0.42		
Lack of time		0.48	
It is too difficult to get out with my child/children		0.59	
I am not interested		0.51	
My child is not interested		0.69	
My spouse/inner circle does not support the activity or is not interested		0.64	
I am inexperienced or insecure about being in nature			0.56
I am afraid someone will get hurt in nature			0.74
I am afraid of animals or insects			0.78

Note. Rotated factor scores reported (Varimax rotation).

**Table 3:** Bivariate correlations among study variables.

	1	2	3	4	5	6	7	8	9
1 Frequency of adult-child nature visits	−								
2 Parental mental well-being (sum)	0.17 ***	−							
Parents’ perceived barriers									
3 Lack of competence and logistics	−0.34 ***	−0.26 ***	−						
4 Lack of time and interest	−0.36 ***	−0.27 ***	0.37 ***	−					
5 Insecurity and fear	−0.13 ***	−0.15 ***	0.33 ***	0.18 ***	−				
6 Age (child)	−0.10 ***	−0.00	−0.08 **	0.10 ***	−0.06 *	−			
7 Gender (child) ^1^	0.04	−0.03	−0.02	0.02	−0.05	0.04	−		
8 Parental educational attainment	−0.06 *	0.09 **	−0.05 *	0.06 *	−0.07 **	0.03	0.04	−	
9 Parents’ perceived economic situation	0.05 *	0.26 ***	−0.15 ***	−0.10 ***	−0.11 ***	−0.02	−0.00	0.31 ***	−
10 Househould type ^2^	−0.08 **	−0.06 *	0.11 ***	0.05	0.02	0.07 **	0.02	−0.06 *	−0.18 ***

Note. Spearman’s rank correlation. ^1^ Child gender (girls = 1, boys = 2). ^2^ Household type (two-parent household = 1, single-parent household = 2). *** *p* < 0.001; ** *p* < 0.01; * *p* < 0.05.

## Data Availability

The data presented in this study are available on request from the corresponding author. The data are not publicly available due to privacy.

## Supplementary Materials

The following are available online at https://www.mdpi.com/article/10.3390/ijerph18136814/s1, Table S1: Regression coefficients for background variables in mediation model.
