# Supplementary material for: Parental Mental Well-Being and Frequency of Adult-Child Nature Visits: The Mediating Roles of Parents’ Perceived Barriers
Source: Int J Environ Res Public Health. 2021 Jun 25;18(13):6814. doi: 10.3390/ijerph18136814 (PMC8297380; doi:10.3390/ijerph18136814)
Supplement: Supplementary file 1 [file ijerph-18-06814-s001.zip › ijerph-1253628-supplementary.pdf]

Supplementary material

Supplementary Table S1. Regression coefficients for background variables in mediation model.

	Lack of competence and logistics			Lack of time and interest			Insecurity and fear			Frequency of adult-child nature visits		
	Unstandardized coefficients (B)	SE	*p-value*	Unstandardized coefficients (B)	SE	*p-value*	Unstandardized coefficients (B)	SE	*p-value*	Unstandardized coefficients (B)	SE	*p-value*
Child age	-0.02	0.01	<0.01	0.02	0.01	<0.01	-0.01	0.01	<0.05	-0.06	0.02	<0.01
Parental educational attainment	-0.01	0.01	0.60	0.05	0.01	<0.01	-0.02	0.01	0.18	-0.10	0.04	<0.05
Parents' perceived economic situation	-0.04	0.01	<0.01	-0.03	0.01	<0.01	-0.03	0.01	<0.01	0.01	0.04	0.84
Household type[1]	0.08	0.03	<0.01	0.03	0.03	0.30	-0.01	0.03	0.75	-0.16	0.10	0.10

Note. Key and background variables included in same model.
[1] Household type (two-parent household = 1, single-parent household = 2).
